# Impact of Deep‐Frying With Different Vegetable Oils on the Nutritional Quality of Grass Carp ( *Ctenopharyngodon idella* ) Fillets

**DOI:** 10.1002/fsn3.70942

**Published:** 2025-09-12

**Authors:** Sara Golgolipour, Ainaz Khodanazary, Kamal Ghanemi

**Affiliations:** ^1^ Department of Fisheries, Faculty of Marine Natural Resources Khorramshahr University of Marine Science and Technology Khorramshahr Iran; ^2^ Department of Fisheries, Faculty of Agriculture and Natural Resources Gonbad Kavous University Iran; ^3^ Department of Marine Chemistry, Faculty of Marine Science Khorramshahr University of Marine Science and Technology Khorramshahr Iran

**Keywords:** deep‐frying, fatty acid profile, grass carp, minerals, vegetable oils, vitamins

## Abstract

The aim of this study was to determine the nutritional value (proximate composition, fatty acid profiles, nutritional quality indices (NQI), vitamins, and mineral contents) of deep‐fried grass carp fillets with different vegetable oils (including olive oil, grape seed oil, and corn oil). The saturated fatty acids (SFAs) of deep‐fried samples with corn oil were significantly decreased, while no significant differences were observed (*p* > 0.05) in the monounsaturated fatty acids (MUFAs) among various vegetable oils. The polyunsaturated fatty acids (PUFAs) content of fillets fried in grape seed oil and corn oil was almost three times that of the raw fillet. The highest and lowest fatty acid contents of the samples were MUFAs and SFAs, respectively. Fillets fried in grape seed oil and corn oil led to a significant reduction in the n‐3/n‐6 ratio. The samples fried with grape seed oil and corn oil exhibited increased PUFA/SFA, UFA/SFA ratios, and hypocholesterolemic/hypercholesterolemic (HH) index. Na content in fillets fried with olive oil was higher than in other treatments. P, Zn, Mg, and K contents have significantly decreased in the fried grass carp fillet. Frying the fillet with different vegetable oils did not significantly alter the Fe content compared to the raw fillet. Ca, Mn, and Cu contents of the grass carp fillet fried with olive oil significantly decreased, while other oils had no significant effect on Ca, Mn, and Cu contents. Vitamin D content in fillets fried in different oils did not differ significantly (*p* > 0/05). Vitamin A content in the raw fillet significantly increased after deep‐frying with different vegetable oils. Deep‐frying significantly reduced vitamins B_1_ and B_3_ content in grass carp fillets (*p* < 0/05). Generally, among all nutritional quality indices (NQI) except the n‐3/n‐6 ratio, fillets fried in grape seed oil and corn oil exhibited optimal lipid quality for human nutrition. In terms of nutrient retention, the data demonstrated that vitamins were best preserved with olive oil, and minerals were best preserved with corn oil.

## Graphical abstract

Effect of diffrenet vegetable oils on the nutritional quality indexes of grass carp.

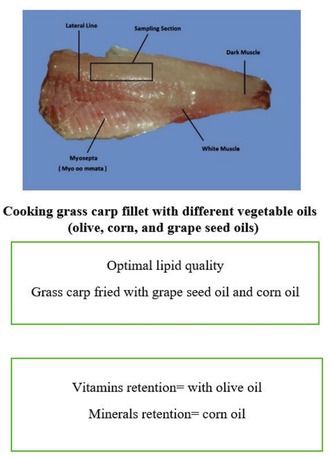

## 1 Introduction

Global fish consumption has risen significantly in recent years due to its recognized health benefits, particularly as an excellent source of complete proteins, ω‐3 PUFAs, and essential micronutrients with high bioavailability (FAO2020). Grass carp ( *Ctenopharyngodon idella* ) is a predominant species in freshwater aquaculture production, widely cultivated and consumed due to its high protein content, favorable fatty acid profile, and abundance of essential micronutrients such as vitamins (e.g., B‐complex, D) and minerals (e.g., selenium, phosphorus). As a lean fish, grass carp is particularly valued for its low saturated fat content and presence of omega‐3 polyunsaturated fatty acids (PUFAs), which are associated with cardiovascular health and anti‐inflammatory benefits (Tocher et al. 2019). Various cooking methods significantly influence the qualitative properties of fish, including texture, flavor, odor, color, and overall acceptability (Ghauomi Jooyani et al. 2011). Cooking meat, particularly frying, leads to the generation of free radicals from lipid oxidation, resulting in changes in flavor and odor (Pérez‐Palacios et al. 2013). However, the nutritional quality of fish fillets is highly susceptible to changes induced by cooking methods. Thermal processing can alter its nutritional profile, including lipid oxidation, protein degradation, and loss of heat‐sensitive micronutrients (Goluch et al. 2021). Although the proper cooking methods could preserve the nutritional composition of fish fillets, such as proximate, vitamin, mineral, and fatty acid composition. Deep‐frying is one of the most popular cooking methods worldwide due to its ability to enhance sensory characteristics such as flavor, texture, and appearance (Mellema 2003). However, this high‐temperature process induces complex physicochemical changes, including protein denaturation, lipid oxidation, and Maillard reactions, which may affect the final nutritional quality of the product (Gökçe et al. 2010). This technique involves immersing food in hot oil, typically between 160°C and 190°C, leading to rapid heat transfer, starch gelatinization, and protein denaturation, which collectively contribute to the formation of a crunchy crust (Yildiz et al. 2024).

The choice of frying oil plays a critical role in these transformations, as oil composition affects heat transfer, oxidative stability, and the absorption of lipids into the food. While vegetable oils (e.g., olive oil, grape seed oil, and corn oil) are frequently used, their varying fatty acid profiles (saturated, monounsaturated, and polyunsaturated ratios) may differentially influence the final nutritional value and safety of fried fish. The selection of olive oil, grapeseed oil, and corn oil over other common frying oils like sunflower or canola oil was due to their unique fatty acid profiles (e.g., high MUFA in olive oil, high PUFA in corn and grapeseed oils), and these differences are expected to impact the final product. The vegetable oil significantly influences lipid oxidation in fried seafood products. Oils with higher polyunsaturated fatty acid (PUFA) content, such as sunflower oil, accelerate the formation of primary (peroxides) and secondary (TBARS) oxidation products during frying compared to more stable oils like palm olein (Chiou et al., 2014). This oxidative degradation reduces the nutritional value of fish fillets by depleting endogenous omega‐3 fatty acids (EPA and DHA) by 25% to 40% after prolonged frying at 180°C (Choo et al. 2018). The use of vegetable oils high in polyunsaturated fatty acids (e.g., sunflower oil) leads to 2–3 times higher peroxide and malondialdehyde (MDA) formation compared to more stable oils like palm olein (Abrante‐Pascual et al. 2024). This oxidation causes a 30%–40% reduction in omega‐3 fatty acids (EPA and DHA) in fish fillets after deep‐frying at 180°C for 8 min (Suryati et al. 2022). Vegetable oil absorption during frying modifies the fatty acid composition of seafood. Studies demonstrate that frying silver carp in sunflower oil increases the ω‐6:ω‐3 ratio from 0.224 (raw) to 5.950 (deep‐frying), potentially altering the food's anti‐inflammatory properties (Zakipour Rahimabadi and Dad 2012). Conversely, high‐oleic canola oil preserves 15%–20% more endogenous EPA/DHA compared to conventional frying oils (Tocher et al. 2019). The development of odor and flavor in cooked products is a complex process resulting from interactions among various compounds, such as secondary products or volatile compounds. Furan compounds play a significant role in forming the distinct aroma of fried products. These compounds (e.g., furan, 2‐furfural, furfuryl alcohol, 2‐pentylfuran, and 5‐hydroxymethylfurfural) are unstable and contribute to a pleasant and desirable aroma. Furans can form from diverse natural precursors in food, including ascorbic acid, carbohydrates, amino acids, fatty acids, and carotenoids. Furthermore, other volatile compounds, such as aldehydes, alcohols, aliphatic hydrocarbons, ketones, pyrazines, pyridines, aromatic hydrocarbons, and esters, play a crucial role in flavor and odor development in fried samples (Pérez‐Palacios et al. 2013). According to our knowledge, several studies have reported the effect of frying on fatty acid profiles and nutrient retention in freshwater fish (Li et al. 2017; Zakipour Rahimabadi and Dad 2012), but vegetable oil‐specific effects on lipid quality indices remain underexplored. We hypothesize that deep‐frying grass carp fillets in vegetable oils with different fatty acid profiles will differentially impact the final nutritional composition, with oils high in monounsaturated fats (e.g., olive oil) better preserving heat‐sensitive nutrients than those high in polyunsaturated fats (e.g., corn oil). Therefore, this study aims to compare the effects of deep‐frying with different vegetable oils on the nutritional value, including fatty acid profile, vitamins, and mineral content of grass carp.

## 2 Material and Methods

### 2.1 Sample Preparation

**FIGURE 1:**
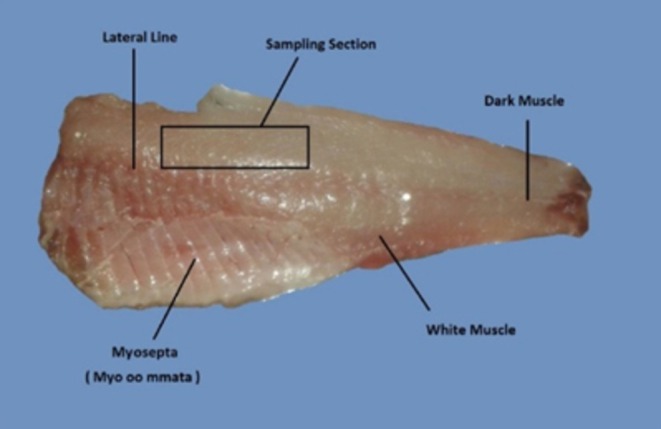
Sampling section of raw and deep‐fried grass carp fillet.

Freshly harvested grass carp ( *Ctenopharyngodon idella* ) were obtained immediately after catching during the harvest season from the Shahid Ahmadian Warmwater Fish Farm (Khorramshahr, Iran). The fish were transported to the processing laboratory at Khorramshahr University of Marine Science and Technology within 2 h of capture, maintaining optimal conditions in polystyrene boxes with ice (fish‐to‐ice ratio of 1:2 w/w). In the laboratory, the fish were rinsed with cold water, decapitated, and eviscerated. Two fillets were prepared from each fish. A 100 g sample from the dorsal region of the lateral line (Figure 1) was selected for cooking according to AOAC Method 16/976 and the seafood processing protocol (Larsen et al. 2010), while another portion was retained as a raw control.

### 2.2 Frying Procedure

Fillet samples were placed in wire‐mesh baskets and fully immersed in preheated oil (180°C) for 5 min. Post‐frying, the baskets were shaken to drain excess oil, and the samples were blotted on absorbent paper. Three oil types were tested: olive oil, grapeseed oil, and corn oil. To avoid oxidative interference, fresh oil was used for each frying replicate. After cooling to room temperature, cooked fillets were deboned and deskinned. All samples were homogenized using a kitchen blender prior to analysis.

### 2.3 Proximate Composition Analysis

Triplicate analyses of cooked and uncooked fish samples were conducted for moisture, protein, lipid, and ash content following AOAC (2002) standards: Moisture content was determined by oven‐drying at 105°C to constant weight. Crude protein content was calculated (6.25 × *N*) from nitrogen measured via the Kjeldahl method. Total lipids were extracted using a Soxhlet apparatus. Ash content was determined by gravimetric analysis after combustion at 525°C for 24 h.

### 2.4 Fatty Acid Profile

Lipid extraction followed the modified Folch et al. (1957) method, with subsequent fatty acid methyl ester (FAME) derivatization. FAME analysis was performed using: Gas chromatograph (Phillips GC‐PU4400) with BPX70 polar capillary column (60 m × 0.32 mm ID, 0.25 μm film); flame ionization detector (FID) at 280°C; injector temperature maintained at 240°C; carrier gas: helium at 1.0 mL/min flow rate.

### 2.5 Vitamin Analysis

#### 2.5.1 Water‐Soluble Vitamins (B₁, B₃)

Quantified by HPLC (KNAUER system) under the following conditions: Detection wavelength: 245 nm; Mobile phase: phosphate buffer/methanol (1000:360 v/v); Flow rate: 1.0 mL/min; Injection volume: 20 μL; Operating pressure: 150–160 bar; Total run time: 22 min (Method adapted from Erosy and Özeren 2009).

#### 2.5.2 Fat‐Soluble Vitamins (A, D)

Analyzed using Thermo Scientific HPLC system with: ODS2 Hypersil column (250 × 4.6 mm, 5 μm); vitamin A detection at 325 nm and vitamin D detection at 265 nm (following the protocol of Stancheva and Dobreva 2013).

### 2.6 Mineral Content Determination

Elemental analysis (Na, K, Ca, Mg, Fe, Mn, Cu, Zn) was performed by acid digestion (HNO_3_); atomic absorption spectrophotometry (GBC Savant AA) (AOAC 2002). Phosphorus content was determined spectrophotometrically at 430 nm after Barton's reagent reaction (Uran and Gokoglu 2014).

### 2.7 Statistical Analysis

All measurements were replicated three times per lot, with results reported as mean values ± standard deviation. Differences among mean values were evaluated using analysis of variance (ANOVA), followed by Duncan's multiple range test at a significance level of *p* < 0.05, employing SPSS version 16.0 (SPSS Inc., Chicago, IL, USA) software. Confidence intervals were calculated consistent with the 95% significance threshold.

## 3 Results and Discussion

### 3.1 The Changes of Proximate Composition

The results of the proximate analysis of raw and fried grass carp ( *Ctenopharyngodon idella* ) fillets using different vegetable oils are presented in Table 1. The fat and moisture content of raw grass carp were 1.78% and 74.33%, respectively. The moisture content decreased significantly in fillets fried compared with raw fillets (*p* < 0.05), which can be attributed to the high frying temperature and thermal denaturation of myofibrillar proteins with water evaporation during heat treatment (García‐Arias et al. 2003; Gladyshev et al. 2006). A similar result in the moisture content of cooked farmed salmon catfish was found by Al‐Saghir et al. (2004). There was no significant difference in the *moisture* content between fried fillets with different oils (*p* > 0.05). Conversely, the fat content significantly increased in fried fillets compared with raw fillets, due to oil penetration as water evaporates during frying (Erosy and Özeren 2009; Koubaa et al. 2012; Rosa et al. 2007). Fillets fried with corn oil had the highest fat content (8.63%). A significant inverse relationship was observed between total lipid content and moisture content in the fish samples (*p* < 0.05). The increased fat retention in fried fillets directly elevated their caloric density (Gokoglu et al. 2004). The protein content of raw fillets was 17.19%. The protein content of grass carp fillets was significantly increased after frying in different oils. This may be due to moisture loss during frying, which concentrates protein mass per unit weight (García‐Arias et al. 2003). The ash content of raw fillets was 1.45%. No statistically significant variation (*p* > 0.05) was observed in the ash content between raw and fried fillets.

**TABLE 1:** Proximate composition of raw and deep‐fried grass carp.

Treatment	Moisture	Fat	Protein	Ash
Raw	74.33 ± 1.85^a^	1.78 ± 0.17^c^	17.19 ± 0.79^b^	1.45 ± 0.05^a^
Fried (Olive Oil)	59.33 ± 4.05^b^	7.88 ± 1.13^ab^	21.69 ± 1.36^a^	1.76 ± 0.48^a^
Fried (Grape Seed Oil)	53.70 ± 0.39^b^	8.63 ± 0.89^b^	20.06 ± 1.10^ab^	1.76 ± 0.28^a^
Fried (Corn Oil)	54.25 ± 0.82^b^	5.86 ± 0.37^a^	19.36 ± 0.58^ab^	1.33 ± 0.06^a^

*Note:* Results are mean ± standard error of triplicates. Means within the same row having different superscripts are significantly different (a‐c) (*p* < 0.05).

### 3.2 Fatty Acid Composition

Table 2 presents the most important fatty acid composition in raw and fried grass carp ( *Ctenopharyngodon idella* ) fillets. In raw fillet fish, the most abundant fatty acids were palmitic acid (C16:0), palmitoleic acid (C16:1), oleic acid (C 18:1) and EPA (C20:5). In raw fillets, oleic acid (C18:1, MUFA) showed the highest concentration among all fatty acids (34.29%), followed by palmitic acid (C16:0, SFA; 22.65%) and linoleic acid (C18:2, PUFA; 8.10%). The relative fatty acid distribution in raw grass carp ( *Ctenopharyngodon idella* ) fillets was MUFA (47.16%) > SFA (28.03%) > PUFA (11.81%). This result is in agreement with Neff et al. (2014) for common carp ( *Cyprinus carpio* ) and Weber et al. (2008) for silver catfish (*Pangasius* sp.). Deep‐frying significantly reduced SFA content (*p* < 0.05), with corn oil showing the most pronounced effect (21.30%). Cooking methods below 150°C showed limited changes in SFAs, while higher temperatures, especially in the presence of oxygen, led to significant SFA reduction (Ghauomi Jooyani et al. 2011). Larsen et al. (2010) and Uran and Gokoglu (2014) confirmed that deep‐fried fillets exhibited the lowest SFA retention. In contrast, Weber et al. (2008) reported increased SFA levels in deep‐frying with soybean and canola oils, but decreased SFA content in hydrogenated vegetable oil systems. The reduction in SFA content observed in fried fillets may be caused by multiple mechanisms: (1) *Lipid exchange* with the frying medium, where unsaturated fatty acids from the oil partially replace SFAs leaching into the oil phase (Dobarganes and Márquez‐Ruiz 2015); (2) *thermal degradation* of short‐chain SFAs (e.g., C12:0, C14:0) at high temperatures (160°C–190°C), generating volatile compounds (Choe and Min 2007); (3) *Maillard reaction*‐mediated binding of SFAs to proteins, reducing extractable lipid fractions (Zamora and Hidalgo 2015); and (4) *Steam distillation effects*, where volatilized water facilitates SFA removal (Nawar 1996). Oleic acid content significantly increased in deep‐frying with olive oil compared to other fried fillets (51.04%; *p* < 0.05). Olive oil is a good source of oleic acid (Portarena et al. 2015). Deep‐fried fillets absorbed olive oil during cooking, which resulted in increased levels of the major oleic acid due to the fatty acid composition of olive oil. This result was similar to findings by Ansorena et al. (2010) and Weber et al. (2008). No significant difference was observed (*p* < 0.05) in MUFA content between raw and fried fillets (*p* < 0.05). Similar studies were performed for white leg shrimp ( *Penaeus indicus* ) by Delfieh et al. (2013). The increase in total PUFA content of fried samples is associated with moisture loss during deep‐frying (Sioen et al. 2006). The results of this study are in agreement with the values presented by Weber et al. (2008), which showed that the fillet fried in soybean oil had the highest PUFA levels. In the current study, the PUFA content of samples fried in olive oil showed a little increase compared to those fried in corn and grapeseed oils. Varela (1988) noted that olive oil has a clustered structure, which inhibits the transfer of frying oil lipids into the food. The n‐6 PUFA content of raw and fried grass carp fillets was significantly higher than n‐3 PUFA. These findings are consistent with those of (Vlieg and Body 1988) and Moradi et al. (2011), who reported that freshwater fish typically contain higher proportions of n‐6 PUFAs compared to marine fish. The predominant omega‐6 fatty acids in the fillets were linoleic acid (C18:2 n‐6) and arachidonic acid (C20:4 n‐6). Similar results were found in studies by (Vlieg and Body 1988) and Moradi et al. (2011), who reported that freshwater fish contain higher levels of omega‐6 polyunsaturated fatty acids (PUFAs) than marine fish. Frying resulted in a significant increase in linoleic acid content. The most important omega‐3 fatty acids analyzed in the fish included α‐linolenic acid (ALA, C18:3 n‐3), eicosapentaenoic acid (EPA, C20:5 n‐3), and docosahexaenoic acid (DHA, C22:6 n‐3). Linolenic acid (ALA, C18:3 n‐3), which is abundant in plant sources, was the predominant omega‐3 fatty acid in grass carp fillets due to the herbivorous nature of this species. There was no significant difference in the EPA and DHA contents between raw and fried samples. However, Larsen et al. (2010) observed a significant decrease in EPA and DHA levels in fried samples.

**TABLE 2:** Fatty acid composition of raw and cooked grass carp fillets.

Fatty acid	Raw	Fried (Olive oil)	Fried (Grape seed oil)	Fried (Corn oil)
C14	2.06 ± 0.04ᵃ	1.92 ± 0.10ᵃ	1.34 ± 0.01ᵇ	1.05 ± 0.01ᶜ
C16	22.65 ± 0.19ᵃ	21.19 ± 0.03ᵇ	17.21 ± 0.06ᶜ	17.13 ± 0.10ᶜ
C18	3.28 ± 0.03ᵃ	3.40 ± 0.03ᵇ	3.82 ± 0.19ᵇ	3.07 ± 0.04ᵇ
C24	0.07 ± 0.01ᵃ	0.06 ± 0.01ᵃ	0.04 ± 0.01ᵃ	0.05 ± 0.01ᵃ
SFA	28.03 ± 0.21ᵃ	26.58 ± 0.11ᵇ	22.43 ± 0.14ᶜ	21.30 ± 0.09ᵈ
C16:1	12.77 ± 0.16ᵃ	11.40 ± 0.37ᵇ	6.85 ± 0.03ᶜ	5.52 ± 0.08ᵈ
C18:1	34.39 ± 12.4ᵇ	51.04 ± 6.48ᵃ	35.85 ± 0.04ᵇ	38.42 ± 0.09ᵇ
MUFA	61.65 ± 18.67ᵃ	62.44 ± 6.76ᵃ	42.70 ± 0.08ᵇ	43.95 ± 0.17ᵇ
C18:2	8.10 ± 0.59ᵇ	9.70 ± 0.89ᵇ	28.82 ± 0.13ᵃ	29.40 ± 0.13ᵃ
C20:4	0.75 ± 0.04ᵃ	0.73 ± 0.04ᵃ	0.64 ± 0.01ᵇ	0.53 ± 0.01ᵇ
∑N6	8.85 ± 0.60ᵇ	10.43 ± 0.93ᵇ	29.93 ± 0.15ᵃ	29.93 ± 0.15ᵃ
C18:3	2.28 ± 0.09ᵃ	2.19 ± 0.12ᵃ	2.38 ± 0.10ᵇ	1.66 ± 0.14ᵇ
C20:5	0.21 ± 0.02ᵃ	0.17 ± 0.01ᵃ	0.21 ± 0.06ᵃ	0.25 ± 0.09ᵃ
C22:6	0.46 ± 0.04ᵃ	0.43 ± 0.06ᵃ	0.43 ± 0.06ᵃ	0.34 ± 0.04ᵃ
∑N3	2.95 ± 0.13ᵃ	2.80 ± 0.19ᵃᵇ	3.03 ± 0.10ᵇ	2.25 ± 0.26ᵃ
PUFA	11.81 ± 0.48ᵇ	13.23 ± 1.13ᵇ	32.97 ± 0.08ᵃ	32.19 ± 0.41ᵃ

*Note:* Results are mean ± standard error of triplicates. Means within the same row having different superscripts are significantly different (a‐d) (*p* < 0.05).

### 3.3 Nutritional Quality Indices (NQI)

#### 3.3.1 PUFA/SFA and UFA/SFA

The ratios of polyunsaturated to saturated fatty acids (PUFA/SFA) and unsaturated to saturated fatty acids (UFA/SFA) are the main indicators for evaluating the nutritional value of seafood (Larsen et al. 2010). The World Health Organization recommended maintaining a PUFA/SFA ratio above 0.4 for optimal dietary health (WHO 2003). A diet with high PUFA/SFA and UFA/SFA has been shown to significantly decrease risks of atherogenesis and thrombogenesis (Fehily et al. 1994). The UFA/SFA index significantly increased in deep‐fried samples compared to raw fillet (*p* < 0.05). Fillets fried in olive oil exhibited a significantly lower PUFA/SFA ratio than those fried in other oils. The observed elevation in both PUFA/SFA and UFA/SFA ratios in deep‐fried samples is likely attributable to oil absorption during the frying process. Weber et al. (2008) found significant differences (*p* < 0.05) in PUFA/SFA and UFA/SFA ratios among deep‐fried silver catfish samples prepared with three oil types: soybean, canola, and hydrogenated vegetable oil. Several studies (Hosseini et al. 2014; Kalogeropoulos et al. 2004; Marques et al. 2010) have reported varying effects of PUFA/SFA and UFA/SFA ratios on lipid metabolism. The results of nutritional quality indices (NQI) are showed in (Table 3).

**TABLE 3:** Nutritional quality indices (NQI) of raw and deep‐fried grass carp (*Ctenopharngodon idella*) with olive oil, corn oil and grape seed oil.

Index	Raw	Fried (Olive oil)	Fried (Grape seed oil)	Fried (Corn oil)
PUFA/SFA	0.41 ± 0.01ᵇ	0.49 ± 0.03ᵇ	1.46 ± 0.00ᵃ	1.50 ± 0.02ᵃ
UFA/SFA	2.09 ± 0.16ᶜ	2.84 ± 0.26ᵇ	3.38 ± 0.01ᵃ	3.57 ± 0.03ᵃ
n‐3/n‐6	0.33 ± 0.03ᵃ	0.26 ± 0.00ᵃ	0.09 ± 0.00ᵇ	0.07 ± 0.00ᵇ
ARA/EPA	3.64 ± 0.58ᵃ	4.17 ± 0.31ᵃ	3.54 ± 1.00ᵃ	2.60 ± 0.75ᵃ
EPA + DHA%	0.59 ± 0.09ᵃ	0.61 ± 0.07ᵃ	0.65 ± 0.00ᵃ	0.59 ± 0.13ᵃ
HH*	1.86 ± 0.18ᶜ	2.70 ± 0.26ᵇ	3.68 ± 0.01ᵃ	3.92 ± 0.01ᵃ
AI**	0.52 ± 0.03ᵃ	0.38 ± 0.03ᵇ	0.29 ± 0.00ᶜ	0.27 ± 0.00ᶜ
TI***	0.75 ± 0.04ᵃ	0.59 ± 0.05ᵇ	0.48 ± 0.00ᵇ	0.48 ± 0.01ᵇ
DHA/EPA	2.15 ± 0.11ᵃ	2.46 ± 0.34ᵃ	2.61 ± 1.04ᵃ	1.56 ± 0.33ᵃ

*Note:* Results are mean ± standard error of triplicates. Means within the same row having different superscripts are significantly different (a‐c) (*p* < 0.05).

* HH = (C18: 1n9 + C18:2n6 + C20:4n6 + C18:3n3 + C20:5n3 + C22:5n3 + C22:6n3)/(C14:0 + C16:0).

** AI = [C12: 0 + 4 (C14:0) + C16:0]/[MUFA = *n*‐3 PUFA + *n*‐6 PUFA].

*** TI = [C14: 0 + C16:0 + C18:0]/[0/5 MUFA + 0/5 (n‐6 PUFA) + 3 (n‐3 PUFA) + (n‐3 PUFA/n‐6 PUFA)].

#### 3.3.2 N‐3/n‐6 and ARA/EPA

The n‐3/n‐6 ratio serves as an important biomedical index. The n‐3/n‐6 ratio showed a significant decrease (*p* < 0.05) in samples fried with corn and grapeseed oils compared to the other samples. In human nutrition, the n‐3/n‐6 fatty acid ratio between 1:1 and 1:1.5 is considered a healthy dietary pattern (Osman et al. 2001). In this study, the n‐3/n‐6 ratio in raw and fried grass carp fillets with olive oil was in the range recommended by WHO. The n‐3/n‐6 ratio in raw and frying fillets with corn oil (0.33:0.07) or grapeseed oil (0.33:0.09) was lower than the recommended limit in the human diet. Gladyshev et al. (2006) reported that the n‐3/n‐6 ratio of raw salmon fillets was approximately seven times higher than in fried samples. In the present study, we observed a decrease in this ratio from 0.33 in raw fish to 0.07 in fried fish samples. This phenomenon may occur through two distinct mechanisms: (1) Thermal degradation of omega‐3 fatty acids during the frying process, and (2) the transfer of omega‐6 fatty acids (particularly linoleic acid [LA, 18:2n‐6]) from the frying oil into deep‐fried fish fillets (Sioen et al. 2006). Delfieh et al. (2013) observed the lowest n‐3/n‐6 fatty acid ratio in fried fish samples. The ARA/EPA ratio is a better nutritional quality index compared to the n‐3/n‐6 ratio due to its greater sensitivity to lipid oxidation (Hosseini et al. 2014). As demonstrated by Larsen et al. (2011), the ARA/EPA ratios correlate with reduced nutritional quality in fish oil. In the present study, deep‐frying significantly reduced the ARA/EPA ratio (*p* < 0.05).

#### 3.3.3 EPA + DHA

EPA + DHA is one of the most critical nutritional quality indices for seafood (Hosseini et al. 2014). The American Heart Association recommended a daily intake of 500 to 1000 mg EPA + DHA to reduce coronary heart disease mortality, achievable through consumption of at least two weekly servings of fatty fish (Hosseini et al. 2014; Larsen et al. 2011; Neff et al. 2014). There was no significant difference in EPA + DHA content between raw and deep‐fried fillets. It indicates that deep frying preserves these essential fatty acids.

#### 3.3.4 HH

The effect of specific fatty acids on cholesterol metabolism was determined by the hypocholesterolemic/hypercholesterolemic fatty acid ratio (HH). The deep‐fried samples exhibited a significantly higher HH ratio compared to the raw fillets. In this study, the HH ratio ranged from 1.86–3.92. Hosseini et al. (2014) and Testi et al. (2006) reported that HH values of different species of fish ranged from 0.25–3.23. Deep‐fried samples with corn oil and grape seed oils were statistically higher in HH ratio compared to fillets fried with olive oil.

#### 3.3.5 AI and TI

Two indices, the Atherogenic Index (AI) and the Thrombogenic Index (TI), were proposed by Ulbricht and Southgate (1991). The nutritional value of lipids (EPA, DHA, etc.) is inversely related to these lipid quality indices (AI and TI) (Ulbricht and Southgate 1991; Hosseini et al. 2014). Hosseini et al. (2014) and Rosa et al. (2007) suggested that a diet with low AI and TI values may reduce the risk of coronary heart disease. In the present study, the AI and TI values of raw fillet were 0.52 and 0.75, respectively. The AI and TI of fried fillets ranged from 0.27–0.38 and 0.48–0.59, respectively. Notably, deep‐frying of grass carp fish resulted in a significant reduction of both AI and TI indices. In various seafood products, the AI ranges from 0.33–2.37, while the TI ranges from 0.01 to 1.18 (Delfieh et al. 2013; Hosseini et al. 2014; Kalogeropoulos et al. 2004; Rosa et al. 2007; Turan et al. 2007).

### 3.4 Vitamin Contents

Table 4 shows the changes in fat‐soluble vitamins A (retinol) and D (calciferol), as well as water‐soluble vitamins B1 (thiamine) and B3 (niacin) of raw and fried grass carp fillets. The vitamin A content in raw fish fillets was 5.50 mg/kg. According to the obtained results, the vitamin A content was higher in fried fillets. Similarly, a study by Erosy and Özeren (2009) and Dias et al. (2003) demonstrated that fried samples retained higher levels of vitamin A compared to raw fillets. There was no significant difference in vitamin A content between oil‐treated fillets (*p* > 0.05). The vitamin D content in raw samples was 0.40 mg/kg. No significant difference (*p* > 0.05) was observed in vitamin D content between raw samples and oil‐treated fillets. Fat‐soluble vitamins exhibited lower sensitivity to heat (Badiani et al. 2013). Conversely, Hosseini et al. (2014) showed that frying reduced the vitamin D content of kutum roach fillets. In their study, Rezaei et al. (2014) reported that thermal processing caused a decreasing trend in vitamin D content in tigertooth croaker. Vitamin B1 and B3 contents of raw fish were found to be 0.80 and 20.80 mg/kg, respectively. Deep‐frying significantly reduced the vitamins B1 and B3 contents compared to raw samples (*p* < 0.05). Similar findings were reported by Hosseini et al. (2014) and Erosy and Özeren (2009), who observed that vitamin B1 content significantly decreased. Among B vitamins, B1 is the least heat‐stable, with thermal degradation being a key factor in its loss during cooking. Additionally, moisture content changes in fillets showed a direct correlation with water‐soluble vitamin levels (Erosy and Özeren 2009).

**TABLE 4:** Vitamin content (mg/kg) of raw and deep‐fried grass carp fillets.

Cooking method	A (Retinol)	D (Calciferol)	B1 (Thiamine)	B3 (Niacin)
Raw	5.50 ± 0.03^b^	0.40 ± 0.01^a^	0.80 ± 0.00^a^	20.80 ± 0.08^a^
Fried (Olive oil)	8.00 ± 0.12^ab^	0.50 ± 0.04^a^	0.50 ± 0.00^b^	6.30 ± 0.08^c^
Fried (Grape seed oil)	9.00 ± 0.07^a^	0.40 ± 0.02^a^	0.30 ± 0.00^bc^	12.50 ± 0.14^b^
Fried (Corn oil)	10.60 ± 0.12^a^	0.20 ± 0.01^a^	0.20 ± 0.00^c^	8.90 ± 0.02^c^

*Note:* Results are mean ± standard error of triplicates. Means within the same row having different superscripts are significantly different (a‐c) (*p* < 0.05).

### 3.5 Mineral Contents

The mineral contents of raw and fried grass carp prepared with different frying oils are presented in Table 5. The sodium content in raw fish fillets was 558.00 mg/kg. There were no significant differences (*p* > 0.05) in Na content between raw and fried fillets, except for fillets fried with olive oil. These results are consistent with findings reported by Marimuthu et al. (2012). Fillets fried in olive oil significantly exhibited higher sodium content (724.67 mg/kg) compared to other treatment groups (*p* < 0.05). This observation is consistent with previous findings by Rezaei et al. (2014), Erosy and Özeren (2009), and Gokoglu et al. (2004). The K content in raw fillets was 1169.3 mg/kg. Deep‐fried fillets significantly decreased the K content (*p* < 0.05), with the following variations depending on the oil used: Grapeseed oil: 724.00 mg/kg; Corn oil: 654.00 mg/kg; Olive oil: 954.00 mg/kg (higher retention than other oils). The Mg content of raw fillets was 144.33 mg/kg. Deep‐frying fillets with olive and grapeseed oils had a significant effect on the Mg content, but not for the deep‐frying fillets with corn oil. Gokoglu et al. (2004) showed that Mg content after frying fillets decreased compared with raw fillets.

**TABLE 5:** Mineral content (mg/kg) of raw and deep‐fried grass carp fillets.

Mineral	Raw	Fried (Olive oil)	Fried (Grape seed oil)	Fried (Corn oil)
Na	558.00 ± 12.70ᵇᶜ	724.67 ± 6.35ᵃ	501.33 ± 24.82ᵇ	571.33 ± 29.44ᵇ
K	1169.3 ± 30.83ᵃ	954.00 ± 47.34ᵇ	724.00 ± 18.47ᶜ	654.00 ± 45.03ᶜ
Mg	144.33 ± 5.48ᵃ	132.00 ± 1.15ᵇ	120.67 ± 2.90ᶜ	138.00 ± 1.05ᵃᵇ
Ca	320.00 ± 48.00ᵃ	157.33 ± 18.66^b^	186.00 ± 6.92^b^	286.00 ± 18.00ᵃ
Mn	0.66 ± 0.09ᵃᵇ	0.48 ± 0.08ᶜ	0.64 ± 0.12ᵃᵇ	0.88 ± 0.13ᵃ
Cu	0.10 ± 0.01ᵃᵇ	0.06 ± 0.01ᶜ	0.13 ± 0.00ᵃ	0.10 ± 0.01ᵃᵇ
Zn	16.40 ± 0.05ᵃ	11.36 ± 0.12ᶜ	13.00 ± 0.21ᵇ	13.28 ± 0.53ᵇ
Fe	13.75 ± 1.44ᵃ	14.00 ± 1.15ᵃ	15.25 ± 0.86ᵃ	22.75 ± 5.77ᵃ
P	2523.20 ± 62.2ᵃ	1496.73 ± 34.26ᵇ	1402.80 ± 71.12ᵇ	1471.71 ± 47.8ᵇ

*Note:* Results are mean ± standard error of triplicates. Means within the same row having different superscripts are significantly different (a‐c) (*p* < 0.05).

The Ca and Fe content of raw fish was 320.00 and 13.78 mg/kg, respectively. There were no significant differences (*p* > 0.05) in Ca content between raw and frying fillet with corn oil. None of the fried fillets had a significant effect on the Fe contents. This result is similar to that of Badiani et al. (2013) and Gokoglu et al. (2004).

The Mn and Cu contents of raw fillets were 0.66 and 0.10 mg/kg, respectively. There was no significant difference in the Mn and Cu contents of fillets between raw and fried samples, except for frying fillets with olive oil. The Zn and P contents of raw fish were 16.40 and 2523.20 mg/kg, respectively. After deep‐frying, Zn and P contents of grass carp were significantly decreased (*p* < 0.05).

## 4 Conclusions

This study evaluated the effects of deep‐frying grass carp ( *Ctenopharyngodon idella* ) fillets with different frying oils (olive, corn, and grape seed oils) on nutritional quality. The findings demonstrate that frying significantly alters the proximate composition, fatty acid profile, vitamins, and minerals, with varying impacts depending on the oil type used. Frying reduced moisture content due to water evaporation but increased fat content via oil absorption, particularly in corn oil‐treated fillets (highest fat retention: 8.63%). Protein content increased after frying, likely due to moisture loss, concentrating protein mass. For optimal nutritional retention, olive oil is recommended for frying grass carp due to its balanced fatty acid profile and mineral preservation. However, grape seed or corn oils may be preferable for PUFA enrichment, albeit with careful consideration of the reduced n‐3/n‐6 ratio. While frying improved some lipid indices (e.g., AI, TI), it compromised heat‐sensitive nutrients (e.g., B1 and B3 vitamins). Nutritional quality assessments of vitamins and minerals revealed superior retention in grass carp fried in olive oil and corn oil compared to other tested vegetable oils. Future research should investigate blended oil formulations (e.g., corn/olive oil composites) and optimized frying parameters (time–temperature combinations) to maximize micronutrient retention while maintaining lipid oxidative stability.

## Data Availability

Data sharing not applicable to this article as no datasets were generated or analysed during the current study.
